# Opportunities, challenges, and contextual supports to promote enacting maturing during adolescence

**DOI:** 10.3389/fpsyg.2022.954860

**Published:** 2022-09-21

**Authors:** Parissa J. Ballard, Lindsay Till Hoyt, Jasmine Johnson

**Affiliations:** ^1^Department of Family and Community Medicine, Wake Forest School of Medicine, Winston-Salem, NC, United States; ^2^Department of Psychology, Fordham University, Bronx, NY, United States

**Keywords:** adolescence, enacting maturity, development, developmental needs, development in context

## Abstract

Conceptions of adolescent “storm and stress” may be tied to a developmental mismatch that exists between young people’s need for meaningful roles and autonomy – which we refer to as a need for *enacting maturity* – and the lack of such opportunities in most adolescents’ contexts. First, we summarize our previous work on enacting maturity, including a review of the key components, links to wellbeing, and the nuances and limitations of this construct. Next, we extend this work by considering how the ecological contexts (e.g., family, school, community) young people are embedded in and their various intersecting social positions and identities (e.g., race/ethnicity, gender, immigrant origin) influence their experiences with enacting maturity. In this section, we pose several key questions for developmental scientists around: (a) identifying a young person’s desire for, and phenomenological processing of, their adult-like roles, (b) understanding how complex and unequal responses to physical maturation shape opportunities for enacting maturity, and (c) attending to disparities in curricular and extracurricular pathways to leadership, responsibility, and autonomy. Finally, we discuss spaces with high potential to support enacting maturity, including both specially designed programs (e.g., youth participatory action research, leadership programs) as well as routine, everyday opportunities (e.g., interactions with teachers, training for companies that employ youth). We offer two levers for supporting enacting maturity across both types of spaces: adult allies and responsive organizations. Looking to exemplary programs, innovative leaders, social media, and case studies, we re-imagine how adults and organizations can promote young people enacting maturing in ways that are safe, worthwhile, and equitable.

## Introduction

Developmental scientists have moved away from characterizing adolescence as a time of “storm and stress,” casting the developmental stage in a negative light, and instead emphasize how the biological and social changes of adolescence give rise to both challenges and opportunities for positive youth development. The overt physical changes that begin with the onset of puberty, combined with advanced cognitive development (Forbes and Dahl, 2010; Blakemore and Robbins, 2012), may make young people look and feel more “adult,” seek opportunities for autonomy and increased responsibility, and enter into new types of relationships with both peers and adults. And yet, the ecological contexts of adolescents rarely scaffold opportunities for maturity commensurate with the explosive growth and development happening in this stage of life. This developmental mismatch (Eccles et al., 1993) is fundamental to understanding adolescent development and may help explain the popular characterization of adolescent “storm and stress” (i.e., parent-child conflict, mood disruption, risky behaviors; Arnett, 1999). In other words, in the absence of developmentally appropriate opportunities for adult-like roles, adolescents might react against family and societal restrictions in the form of parental conflict, poor mental health, and/or unhealthy risk behaviors. In this manuscript, we join with others calling for new ways to characterize, understand, and ultimately support adolescent development going beyond “storm and stress” by re-focusing on creating positive developmental opportunities to harness the normative changes of adolescence and help young people thrive.

We seek to understand the ways in which young people experience meaningful roles and autonomy – which we refer to as enacting maturity – during early and middle adolescence. First, we summarize our previous work on enacting maturity (Hoyt et al., 2021), including a review of the key components, links to wellbeing, and the nuances and limitations of this construct. Next, we extend this work by considering how the contexts young people are embedded in and their various intersecting identities influence their opportunities and experiences with enacting maturity. Finally, we showcase existing settings for enacting maturity and re-imagine how adults and organizations can promote young people enacting maturing in ways that are safe, worthwhile, and equitable.

### Defining and measuring enacting maturity

Based on extensive research documenting the shifting developmental needs young people have for autonomy and contribution during early and middle adolescence (e.g., Eccles et al., 1993; Ryan and Deci, 2000; Crone and Dahl, 2012; Nelson et al., 2016), we proposed the concept of *enacting maturity* as a helpful way to conceptualize the extent to which early and middle adolescents perceive experiencing adult-like roles and autonomy in their daily lives (Hoyt et al., 2021). Drawing on ideas of developmental mismatch (e.g., Eccles et al., 1993; Piquero and Moffitt, 2005), self-determination theory (Ryan and Deci, 2000), and tenets of positive youth development (e.g., Lerner et al., 2005), we aimed to test whether the lack of developmentally relevant opportunities for young people to enact maturity (and thus meet their developmental needs) could lead to poor psychological wellbeing and problem behaviors. Although many existing survey measures capture related constructs, for example autonomy and decision-making opportunities, communication with adults, responsibility, and leadership, most are context-specific (e.g., in family, school, or community) and we did not find a measure assessing the extent to which young people enact maturity across the contexts of their everyday lives (see Hoyt et al., 2021 for a review). This led us to develop a new measure in a multi-phase project.

Guided by a literature review and drawing on data from focus groups and surveys with middle school and high school students, we created and validated a survey measure with four related but distinct (*r*s = 0.28 −0.54) dimensions of enacting maturity: independence, responsibility, communication, and leadership. *Independence* refers to autonomy in making choices over a wide range of contexts (e.g., phone/computer use, food choice, bedtime), as well as increased opportunities to stay home or go out without the presence of adults. *Responsibility* encompasses both personal care (e.g., taking ownership for their own belongings, doing homework without being asked) and family duties (e.g., helping out around the home). We also uncovered a *communication* factor, specifically defined as the ability to navigate discourse, debate, and serious conversations with adults about important topics. The final element, *leadership*, includes holding a specific role in one’s school or community, as well as a general sense of social status.

As expected, both responsibility and communication were associated with significantly lower levels of problem behaviors, including substance use and risk taking behaviors; links between leadership and wellbeing were not statistically significant. Independence, on the other hand, was associated with *more* substance use and risk taking and increased levels of perceived stress. Too much independence could induce stress and/or serve as a proxy for stressful environmental factors that could increase risk for problem behaviors. For instance, youth may be home alone or left to make choices independently not because their parents/guardians are responsive to their developmental needs for autonomy and respect, but because the adults in their homes are not able to be physically present, leaving young people with excess unstructured and unmonitored time (Laird et al., 2008). Similarly, too much or certain forms of responsibility may also be a burden for young people. For example, the responsibility dimension of the enacting maturity scale did not capture daily caretaking, financially supporting your family, or language/cultural brokering. Independence or responsibilities that precede developmental readiness may demand too much maturity (Johnson and Mollborn, 2009; Kendig et al., 2014). Further, like all dimensions of enacting maturity, there may be important differences by context and culture. Our research is based in the United States, a Western context for adolescent development with a cultural emphasis on independence, although with many variations in subcultures.

Conceptualizing and measuring enacting maturity is a step toward understanding how adolescents perceive opportunities to enact maturity in their everyday lives. However, it is important not to romanticize the idea of enacting maturity and to acknowledge nuances and potential costs of autonomy for youth depending on their contexts and backgrounds. Contextualizing our understanding of enacting maturity will help developmental scientists focus on adapting or creating supportive environments where adolescents from diverse racial/ethnic, gender, and socioeconomic backgrounds can enact maturity in developmentally promotive ways.

### Enacting maturity in context: Key questions to guide future research

As described in Spencer’s (1995, 2006, 2008) phenomenological variant of ecological systems theory (PVEST), environmental risk and protective factors are not deterministic; youth experience them as supports or stressors depending on their identities and ecological contexts. Aligned with Velez and Spencer (2018), we believe that whether and how enacting maturity promotes wellbeing and/or confers developmental risk depends on both the contexts youth are embedded and how they make sense of their experiences (i.e., their phenomenological processing; Velez and Spencer, 2018).

Adolescents are developing within multiple intersecting contexts. They are embedded in interlocking systems of privileges and oppression across multiple domains (e.g., race/ethnicity, gender, socioeconomic position, sexuality; Collins and Bilge, 2016; Crenshaw, 2019). Velez and Spencer (2018) remind developmental scientists to examine how social positions, identities, and oppressive conditions combine to create unique experiences for each individual. They integrate PVEST and intersectionality perspectives to understand identity development and argue that “adolescent outcomes should be understood both from the top and the bottom, including how youth interpret and cope with their vulnerability, based upon experiences of interlocking systems of oppression” (pg. 76). Indeed, with increased capacity for complex social cognitive functioning during the second decade of life, adolescents become capable of deep meaning-making processes, which allow for an advanced level of reasoning (Spencer et al., 1997). Thus, youth’s everyday experiences are shaped by broader power dynamics, their own identities and social positions, and how they make sense of their experiences.

Drawing on this integrated framework, we examine the concept of enacting maturity through the lens of intersecting social positions, identities, and ecological contexts that shape opportunities and experiences of enacting maturity. In this section, we explore three issues related to disparities for enacting maturity: choosing versus needing to enact maturity, differences in how society responds to maturing adolescents and subsequent restrictions on enacting maturity, and disparities in formal opportunities to enact maturity. In each section, we offer key questions to guide future research on enacting maturity.

#### Choosing vs. needing to enact maturity

Although the growing desire for adult-like roles and responsibilities is recognized as a normative developmental process across adolescence (Eccles et al., 1993; Yeager et al., 2017), it is also important to recognize that many young people are *required* to fulfill mature roles, some perhaps before they are ready. Rather than experiencing a mismatch in desired autonomy within restrictive environments, some youth may face a reverse mismatch in having to take on mature social roles before they are developmentally prepared. Desiring, seeking, and choosing opportunities for enacting maturity involves having discretionary time and the latitude to accept or decline adult-like roles and responsibilities. Thus, there may be an underlying assumption of privilege in developmental concepts related to increasing need for independence, responsibility, and the desire to enact maturity when in fact many young people are required to enact maturity as a matter of survival, need, or familial and societal expectation. For example, two young people may apply for an afterschool job for disparate reasons: One teenager may *want* extra spending money, another may *need* to financially support themselves or their families.

Familial expectations require some young people to enact maturity *via* caretaking responsibilities for younger or elder family members (Fuligni et al., 1999; Jurkovic et al., 2004; Mangeli et al., 2017). Familial role expectations vary across cultural backgrounds in the United States, with some cultures, such as Asian and Pacific Islander (AAPI) and Latinx, emphasizing communalism and interdependence over individualism (Harrison et al., 1990; Chao and Tseng, 2002; Christophe and Stein, 2022). In many households, AAPI and Latinx youth are expected to spend a significant amount of time with their families and to provide emotional, financial, and instrumental support to family members. Teens from families newer to the United States, in particular, experience an increase in adult-like role expectations of language/cultural brokering especially when parents or guardians work long hours outside the home and face language barriers (Fuligni et al., 2002; Suárez-Orozco and Qin, 2006). Familial role expectations may also vary by gender, with young women expected to assume more care-taking roles compared to young men in many cultures (Sharma et al., 2016). Girls in immigrant families report more responsibilities compared to their brothers (Valenzuela, 1999; Ginorio and Huston, 2001; Williams et al., 2002) with duties including translating; advocating in financial, medical, and legal transactions; cooking and child-care responsibilities; and acting as surrogate parents (Valenzuela, 1999; Suárez-Orozco and Qin-Hilliard, 2004). Some find meaning to such roles, for example through increased personal and interpersonal competence (Jurkovic et al., 2004) or a sense of identity and purpose while they are in the process of adapting to American society and culture (Fuligni and Pedersen, 2002). However, such roles may also be burdensome, providing undue feelings of familial responsibility that restrict role exploration in other domains. Both the context of obligations and the phenomenological experiences young people have with their obligations may shape development.

Finally, these pressures and disparities may be exacerbated by familial events (e.g., parental job less) and sociopolitical events (e.g., recession, public health crises), particularly for young people exposed to economic and social insecurity. The COVID-19 pandemic revealed these acute disparities in the *need* to enact maturity, as financial and social strains placed a higher burden on families with fewer resources. As schools shifted online, some youth were forced to juggle adult roles and responsibilities such as teaching and looking after younger siblings, caring for elders, cooking and other household chores, and “essential work,” while also adapting to their experiences in virtual school. As one young person explained, “*I have my little brother, who was getting work, but it was just papers sent home with no guidance or help from teachers. So, I had to teach my little brother because my parents aren’t really good with English, so they can’t really translate what they know in Kurdish into English, so I have to do all the teaching for him”* (16 year-old girl; Shah et al., 2021). This quote helps illustrate how a teenage girl from an immigrant family *needed* to step into a new, mature role, during an especially challenging time when it was difficult for anyone to take on additional responsibilities.

Reflecting on these themes, key questions to consider are: To what extent is enacting maturity driven by choice versus need, and what are the potential implications for developmental trajectories? On the one hand, teens who must enact adult-like roles may not benefit as much from these roles if they constrain life prospects, relationships, and growth. On the other hand, even mature roles that arise from obligation might be meaningful for young people and fulfill developmental needs for autonomy and responsibility, if youth feel capable and supported. Following PVEST, it will be instructive for empirical research on enacting maturity to consider both how the structural realities (i.e., conditions that constrain choices and create needs for adolescents to adopt mature roles) and how the phenomenological experiences adolescents have in those roles (i.e., how they make meaning of their various roles) shape development. Qualitative approaches will be particularly useful to understand these complex interactions.

#### Complex and unequal responses to maturation shape opportunities

While young people may expect increased authority to make decisions about their schedules, appearance, health, and relationships as they get older, many identity and contextual factors may determine if, when, and for whom enacting maturity is safe and beneficial. The physical changes of adolescence, especially related to puberty, provoke new social challenges as youth are often perceived and treated differently by peers, parents, teachers, or other adults. However, the implications and consequences of appearing or acting more “adult” differ for young people based on many factors such as race/ethnicity and gender (Goff et al., 2014; Blake and Epstein, 2019; Hlass, 2020). In turn, parents and other adults may respond to this reality in ways that shape future opportunities for enacting maturity. In this section, we highlight how adolescents’ advancing development elicits complex responses and unequal opportunities for enacting maturity tied to one’s intersecting social identities and context. We discuss how this process plays out for Black/African American adolescent boys, transgender young people, and youth from Latinx and Filipina immigrant families. Although these examples are not exhaustive, they showcase timely issues in both public and academic spaces.

Youth of color experience societal expectations, biases, and discrimination (Spencer et al., 2003) that shape both their experiences of maturing and their opportunities for enacting maturity. For example, adults and youth associate being Black and male with low intelligence, hostility, and violence (Blaine and McClure Brenchley, 2007). Although these stereotypes are already evident in childhood, pubertal development may trigger further negative stereotypes for boys of color who begin to “look like a man” (e.g., Deardorff et al., 2019) and Black boys are more likely to be mistaken as older and less innocent compared to their White same-age peers (Goff et al., 2014). This is illustrated in research as well as comments from officials responsible for public safety. For example, after 12-year-old African American Tamir Rice was killed by police, the President of the Cleveland Police Patrolmen Association explained *“Tamir Rice is in the wrong. He’s menacing. He’s 5-feet-7, 191 pounds. He wasn’t that little kid you’re seeing in pictures. He’s a 12-year-old in an adult body”* (Quote from Schultz, 2015). Thus, societal perceptions may contribute to anti-Black police violence, including the killing of unarmed Black boys shot by police in the United States (Wilson et al., 2017). In addition to experiencing violence, Black teens experience disproportionate school suspensions, arrests, and being prosecuted in adult court (Marchbanks and Blake, 2017; Hill, 2018). Black adolescents perceived by others to be older, reported more anger than same-aged Black boys with “babyfaces,” perhaps prompting physically advanced boys to be defensive in these contexts because they face greater perceptions of threat (Stevenson et al., 2002). The reality of structural and interpersonal discrimination and prejudice may prompt “restrictive” parenting practices aimed to protect certain youth (Copeland-Linder et al., 2011; Dotterer and Lowe, 2015; Hill, 2022). Racial-ethnic socialization (i.e., the developmental process of sharing and fostering cultural heritage and history and promoting ethnic pride within a family) often includes preparing youth to encounter and combat the racial biases and gendered racial stereotypes that threaten their lives (Hughes et al., 2006). Many Black and Latinx parents have “the talk” with their children about how to safely conduct themselves when interacting with police officers or other adult authority figures (Fox-Williams, 2018). In many ways, youth of color are stripped of opportunities to enact maturity due to racism in their everyday settings. For example, youth may be taught not to take risks that might be positive or normative (Duell and Steinberg, 2019) such as *not* to defend themselves (e.g., stay silent if a teacher reprimands them for something they didn’t do) or *not* to speak up about injustice (e.g., avoid protests or demonstrations) because the consequences for them are likely to be greater (Center for the Developing Adolescent, n.d.). Parents may also restrict their teens’ clothing choices (e.g., hoodies, hats, gang-affiliated colors) and limit when and where they socialize (e.g., parental monitoring, curfews). In Black and Latinx families in particular, parents and youth are often forced to make choices that center safety and survival over individual autonomy. In addition to societal reactions shaping subsequent opportunities for enacting maturity, experiences with discrimination and racism can affect young people’s mental health and ability to understand and make meaning of their experiences thus illustrating complex interactions between structural contexts and phenomenological processes outlined by PVEST.

Transgender and gender diverse (TGD) and lesbian, gay, bisexual, or queer/questioning (LGBQ) youth also have to navigate stigma and discrimination in their environments, which can affect parent-child relationships and negotiations about diverse aspects of enacting maturity including appearance, dating, extracurricular activities, and healthcare choices. From legislation (e.g., “Don’t Say Gay bill”; Diaz, 2022) to restrictions in participating in sports teams and using the bathrooms for the gender they identify with, TGD youth are regularly excluded or marginalized in developmental spaces (e.g., school, extracurricular activities). These measures create a climate where school may not feel like a safe space for young people to enact maturity through normative activities during adolescence such as debating issues with peers and teachers, asking out a classmate, or organizing a club. TGD youth may also face barriers to enacting maturity in their own bodies such as restricted access to gender-affirming care including the use of hormones to delay puberty and to promote physical development that is consistent with a young person’s gender identity (Conron et al., 2022). Although the majority of transgender adolescents maintain their gender identity into adulthood (Olson et al., 2022), many parents, lawmakers, and medical professionals think that adolescents are not mature enough to make these decisions. Before most *trans* individuals consider surgery or hormones, many seek a *social transition*, in which they change their gender expression to match their gender identity. While the social transition itself *does not* harm TGD youth, and indeed may result in better mental health outcomes (Olson et al., 2016; Durwood et al., 2017; Rae et al., 2019), harassment and bullying based on gender identity from classmates, teachers, and school staff remains a threat to safety and wellbeing (Turban et al., 2021). Therefore, even when parents support their TGD child’s gender identity, they may monitor, discourage, or restrict gender-affirming social transitions choices (e.g., haircuts, make-up, clothing) for fear of bullying or victimization. Additionally, youth may restrict their own style, behavior, or expression due to societal pressures to protect themselves from mental or physical harm.

In response to their child’s growing orientation toward peer groups and spending more time outside the home, as well as their emerging sexuality, some parents may become *more* concerned for their child’s safety and potentially *more* restrictive during early or middle adolescence. Importantly, these parent-child relationships and negotiations are entrenched in cultural values that intersect with gender (Suárez-Orozco and Qin, 2006; Chao and Kanatsu, 2008). Across many ethnic backgrounds and historical contexts, there is evidence of stricter parental control of immigrant adolescent girls compared to boys. For example, Latinx boys are granted greater autonomy as they transition from childhood to adolescence, a greater range of mobility, and more freedom to explore contexts outside of the home compared to girls (Crockett and Russell, 2013; Domenech Rodríguez et al., 2009). In research focused on Filipina Americans, Espiritu (2001) found that many children talked about a “double standard” in their families, related to parental control. When questioned about this double standard, parents explained that “girls are different;” for example, one Filipino parent said: *“I have that Filipino mentality that boys are boys and girls are different. Girls are supposed to be protected, to be clean. In the early years, my daughters have to have chaperons and curfews. And they have to be virgins until they get married. The girls say that is not fair. What is the difference between their brothers and them? And my answer always is, “In the Philippines, you know don’t do that. The girls stay home. The boys go out.” It was the way that I was raised. I still want to have part of that culture instilled in my children.”* (Espiritu, 2001, p. 430). Across many studies, immigrant girls may not be allowed to go to parties, date, work outside the home, participate in extracurricular activities, or wear the clothing or makeup that they want to. Although the literature suggests that there may be some positive implications of strict parental control, such as educational success (Fan and Chen, 2001), many immigrant girls face clear barriers to enacting maturity.

Altogether, this leads to another key question to consider: How do societal reactions to maturing young people, based on their identities and backgrounds, influence their opportunities to enact maturity? Future research can ask youth about how they think adults perceive them as well as potentially documenting societal reactions and perceptions of youth (e.g., by analyzing media messages about youth that they are exposed to or by surveying adults in proximal environments about their perceptions of youth). Further, how do disparate societal reactions to maturing adolescents, and subsequent constraints and opportunities, affect positive development among young people?

#### Disparities in formal opportunities to enact maturity

In addition to the inequalities that arise due to people’s reactions to physical maturation across various dimensions of identity, the formal opportunities young people have for enacting maturity are also unequal. Formal opportunities differ for groups of youth across many sociodemographic characteristics such as socioeconomic position, race/ethnicity, gender, and age. As an example, one powerful opportunity for enacting maturity during adolescence is through civic engagement, or joining with others to address issues of collective concern in communities. Young people from lower socioeconomic backgrounds and racial/ethnic minority backgrounds are offered fewer and lower-quality civic opportunities (Kahne and Middaugh, 2008; Levinson, 2010; Gaby, 2017). As one young person from a civically underserved community put it, “We all have ideas how things could be better in our schools. We’re just waiting for someone to ask us. No one ever asks us our opinion.” (Generation Citizen, 2018). The types of activities available to youth, from organizing to school clubs to 4-H programs (i.e., a large youth development organization in the United States), also differ across settings. Online spaces have potential to increase opportunities for youth voice and contribution (e.g., Wilf and Wray-Lake, 2021); however, young people still have unequal access to resources such as technology that would allow them to utilize civic opportunities in these spaces. Even where civic opportunities are available, young people don’t have equal access to transportation or free time (e.g., if they need to work a paid job) for civic participation.

As implied by PVEST, it is important to consider not only the unequally distributed structural opportunities for enacting maturity but the varying experiences young people have when they do participate and how they make meaning of these experiences. For example, civic experiences online and offline vary across social positions and identities. Certain forms of civic engagement such as activism can present both physical and psychological risk to young people from racial/ethnic and sexual minority identities as well as immigrant backgrounds (e.g., Santos and VanDaalen, 2018; Maxie-Moreman and Tynes, 2022). In the context of opportunity and experiential disparities, civic engagement may provide an important route to positive development *via* enacting maturity especially for young people traditionally left out of civic life. Depending on the phenomenological experience young people have, scaffolded opportunities to make meaningful connections between civic issues and young peoples’ lives *via* civic engagement can allow young people to develop complex sociopolitical understanding, connect with others with shared values, find support, develop skills and support wellbeing.

Therefore, a final set of key questions for research on enacting maturity include: How can we make formal opportunities more equitable across youth? Further, is it developmentally ideal to have opportunities for enacting maturity across multiple contexts, or is it sufficient to have such opportunities in one context (e.g., school, or home, or community)? If the latter, we must consider prioritizing opportunities for enacting maturity in public spaces (e.g., school, neighborhood programs) for youth who do not have the ability to enact maturity at home, or conversely encouraging parents/families to promote opportunities to enact maturity at home for youth who have fewer community or school resources. There are also individual differences (e.g., introversion vs. extroversion) that may play an important role in finding the most appropriate context(s) for enacting maturity. Overall, in spaces that provide opportunities for enacting maturity, it is critical that young people can experience maturity in authentic and safe ways. Moving away from deficit models of adolescence, and amplifying underrepresented voices, future researchers should consider participatory approaches to answering these questions, including youth participatory action research and/or youth advisory boards (described in more detail below).

### Re-imagining contexts for enacting maturity: The role of adult allies and responsive organizations

In this section, we discuss ecological contexts with high potential to support enacting maturity. Although our focus is on promoting youth development, we join with researchers and practitioners calling to “focus change efforts on transforming not just young people, but the adults that work with youth at the point-of-service as well as implementing institutional and systemic change” (Zeller-Berkman, 2019, p. 11). To that end, we offer two levers to support enacting maturity: adult allies and responsive organizations. We review examples of specially designed programs (i.e., participatory models, leadership programs and positions) and everyday opportunities (e.g., interactions with teachers, experiences on social media) to support enacting maturity. Returning to the framework of PVEST, much of adolescents’ phenomenological processing (i.e., meaning-making; Velez and Spencer, 2018) happens within their proximal developmental contexts. Within both formal and informal settings, adult allies and organizational policies and structures can scaffold positive opportunities for independence, responsibility, communication, and leadership.

#### Adult allies

Research and praxis in education, psychology, and public health offer guidance for how adults within youth settings can empower youth *via* developing youth-adult partnerships. Adult allies are the adults in youth settings who hold power and who gain skills to share their power and amplify youth voices (Zeldin et al., 2013; Richards-Schuster and Timmermans, 2017). Adult allies could be aware of structural barriers and opportunities for various youth and advise them on meaningful roles that would fit into their lives (connectors), could use their power to amplify youth opinions and needs (advocates) and could help young people reflect on their phenomenological experiences in different roles (advisors).

Adults within formal contexts (e.g., schools, religious organizations, clubs, workplaces) who work with youth can become allies to young people with intentionality, for example by seeking training (Morrel-Samuels et al., 2014) and working to balance power between adult and youth partners (Li and Julian, 2012; Zeldin et al., 2013). Formal adult ally training is available to individuals and organizations implementing specific programs such as Youth Empowered Solutions (Training: Yes, n.d.) and Dover Youth to Youth (Youth 2 Youth, n.d.). Other organizations offer training for adult allyship for specific groups of young people (e.g., TGD or LGBQ youth) such as the Trevor Project (Ally Training, n.d.) and many online resources summarize general best practices for being an ally to young people (Adult Trainings – UpRISE Youth Tobacco Movement, n.d.; National Sexual Violence Resource Center, 2014). Adult allies can connect young people to existing opportunities for enacting maturity by helping youth align their interests, skills, and life circumstances with particular opportunities to enact maturity. In one program called Authoring Action, young people described how the adult leaders connect them to the people and resources they need to pursue jobs and projects such as creating films (Ballard et al., 2021). Drawing on their deep knowledge of their community and the young people they work with, the adult leaders of this program exemplify the connector role that adult allies can play to create opportunities for enacting maturity.

In the spaces where adults have power, they can advocate for the opinions and needs of young people, or better yet, invite young people “to the table” to share power. There are numerous examples of organizations implementing different models to incorporate youth voice. One example of creatively inviting young adults to share power is an initiative at Wake Forest University where students are in charge of purchasing art for campus buildings with university funds (McGrath, 2021). This simple initiative recognizes college students as both the consumers of art at their university and as having necessary expertise about art and their peers. Another arts-based initiative, the Mural, Music and Arts Project in East Palo Alto, California, commissions teens and young adults to create murals *via* a paid program that focuses on youth skill-building and city beautification (Taaffe, 2017). In both examples, young people are invited to share power, entrusted to make consequential decisions, and supported with the funding to do so.

Outside of formal program settings, adult allies can look for ways to increase the number of opportunities for enacting maturing in everyday contexts, as well as helping them make meaning of their experiences as advisors. Teachers who are adult allies may effectively incorporate student input in lesson planning, and respond to students’ needs by tailoring assignments based on evolving interests. They can shape the phenomenological experiences young people have by helping students see how their work is important, how their maturity helps class and school functioning, and how adults often need young people to “rise to the occasion.” Adult allies can help young people recognize the contexts where they are able to enact maturity and where they are not. Further, adult advisors can help young people make sense of such contradictions, and to make meaning of their own experiences of enacting maturity in family and work contexts, regardless of the reason being necessity or choice, in a way that supports youth autonomy and sense of purpose, and emphasizes their maturity. This may be through helping young people reflect on how their responsibilities at home help contribute to their family’s functioning (Fuligni et al., 1999) or how taking responsibility for school work is connected with their future goals. Further, adult allies can support youth mental health by noticing, and naming, the costs of racism and other oppressive experiences.

Finally, adult allies can help create both physical and online spaces where young people feel safe, connected, listened to, and empowered as leaders. Based on their recent research examining Black youths’ experiences with racism, stress, and safety, researchers argue that youth need more spaces that are truly youth-centered and youth-run without barriers such as cost, permits, or needing a specific purpose to use the space. The researchers further called for leaders working with youth to listen to young people’s needs and voices (Ortega-Williams et al., 2022a). As Dr. Ortega-Williams described in an interview with the Society for Research on Adolescence “we want to have deliberate investment in youths’ resources and spaces. Also, as adults, we can transform spaces. We can make sure we’re not harming young people” (Ortega-Williams et al., 2022a). Social media is another informal setting where youth spend time and, when done in the right way, adults can show up as allies or partners with youth. As an example, while politicians are notoriously unresponsive to young people (e.g., Shea, 2009), some, such as Rep. Christina Haswood (Okeson-Haberman, 2020), have been successful in effectively partnering with young people and inviting them into political discussions (e.g., through TikTok and Instagram Live). Gaining skills in how to best communicate with and empower young people in adult-led physical and online spaces (as connectors, advocates, and advisors), and creating more youth-led spaces, are complementary ways for adult allies to support equitable and safe avenues for enacting maturity.

#### Responsive organizations

Beyond individual adults becoming allies to young people, youth-serving organizations can promote or hinder young people’s ability to enact maturity. Organizations operate within overarching, institutional systems of oppression and privilege (e.g., laws, norms), but they also have their own specific policies and structures that could expand adolescents’ everyday opportunities to practice adult-like roles and responsibilities. However, the organizations where young people spend a lot of time, such as schools, afterschool programs, sports, jobs, and religious institutions, are often not designed, or lack the capacity, to support enacting maturity.

Participatory models are a promising approach for youth-serving organizations and programs. Participatory models are diverse, but “share an assumption that youth have unique expertise that is needed for understanding and addressing key issues affecting their health, development and wellbeing” (Ozer et al., 2020 pp. 4). Two such models include Youth Participatory Action Research (YPAR) and youth advisory boards. These models vary considerably in level of engagement and the role of youth, and the exact nature of the participatory model will vary depending on the settings (Suleiman et al., 2021). In the proximal settings of schools, some teachers implement YPAR projects in the classroom (Mirra et al., 2015; Rubin et al., 2017). Some teams are investigating specific proximal settings outside of school, for example, one project employs a YPAR approach to understand the needs of children of incarcerated parents (Abraczinskas et al., 2022). In addition to participatory research models, youth-serving organizations and initiatives may employ youth advisory boards to advise their work. Youth advisory boards can be used across settings, for example in foster care (e.g., California Youth Connection, n.d.), clinical care or patient settings (Teen Advisory Council- John Hopkins Hospital, n.d.), research (e.g., Ortega-Williams et al., 2022b). Youth advisory boards may also be crafted around specific community issues, such as substance misuse prevention (e.g., CADCA’S National Youth Advisory Council, n.d.) or policing (e.g., Youth Advisory Council - Howard County, n.d.). At a more distal level, some funding agencies are prioritizing funding for projects that partner with youth have allocated resources to understand how to evaluate the capacity and potential for these research partnerships (Suleiman, 2021).

Outside of formal partnerships or programs, there are also many things in adolescents’ everyday lives that offer them increasing opportunities for enacting maturity as they take on more ownership over their learning, their belongings, and their time. Any youth-serving organization, or company that employs youth, can take advantage of this developmental shift by supporting young people in taking on more responsibilities. Indeed, many companies hire teenagers as young as 14-years-old (e.g., fast food restaurants, retail or grocery stores, summer camps) and represent an important and underutilized setting for enacting maturity. Although performing paid work in and of itself does not necessarily provide positive experiences for growth and autonomy. However, getting youth employees involved in mentoring or training others, decision-making, and advancement opportunities could promote more positive outcomes for both youth and companies. In order to do this successfully, these companies should seek out and reward adult allies who support, collaborate with, and check-in on their teenage colleagues.

Relatedly, many companies advertise internships for adolescents and young adults that are specifically designed to enhance elements of enacting maturity (e.g., responsibility, leadership, communication) and provide job-specific skills that are a key stepping stone to career success. However, given that most internships are unpaid, these opportunities may further exacerbate long-term disparities between youth from more privileged backgrounds (who can afford to work for free) and youth who face more social and economic stress. There are many excellent national and local youth programs that scaffold and train youth from marginalized backgrounds to build skills and explore career options. For example, YouthBuild is a program working with “opportunity youth,” (i.e., youth between the ages of 16 and 24 who are neither in school nor employed), to help them build skills, connect them with opportunities, and harness their untapped potential to improve their communities (Why YouthBuild, n.d.). Harlem Children’s Zone’s Employment and Technology Center is a 4-year program that provides youth with in-depth knowledge and real world experience with the STEM field (The Employment and Technology Center, n.d.). However, it is also vital that large companies who employ youth integrate some of these priorities into their hiring, training, and internship programs.

Including young people on governing bodies is another direct way to engage adolescent stakeholders in the organizational system that serves them, and also provides an underrepresented and unique point of view that can improve organizational outcomes. In a school setting, for example, students bring inside perspectives on school policies (e.g., dress codes and class schedule), budget priorities, and academic and school climate issues (e.g., shifting to competency-based learning, disciplinary practices, equity). One study reported that 19 states included at least one student member on their state boards of education (Fletcher and King, 2014). A high school in on of these states, New Hampshire, found that since the school board adopted some of these youth-informed approaches, the school’s dropout rate decreased by more than half, from 3.9 percent in the 2010–2011 school year to just 0.6 percent in the 2016–2017 school year (Minero, 2017; Benner et al., 2019). There is little precedent, and complicated legal issues, for considering youth governance in youth-serving organizations such as private/public companies, local government, or non-profit organizations (Chan, 2012). However, membership on a board of directors is not the only option for an organization to reap the value and benefits associated with youth leaders. Other ideas include: forming a youth advisory board, inviting youth to join a specific committee or leading projects that utilize their perspective, establishing mentor-mentee pairings between youth and organizational leaders, or having the youth participate in board meetings as a non-voting guest (Chan, 2012).

## Conclusion

Throughout history and across cultures, young people begin to take on more mature roles and responsibilities across the second decade of life. At the same time, families, communities, and governments are charged with protecting children and adolescents and helping usher them safely into adulthood. The mismatch that can result between a young person’s growing desire for mature roles and experiences, and a general lack of opportunities to fulfill these developmental needs, may contribute to the characterization of adolescence as a time of “storm and stress” as it can result in tensions between youth, parents, and society. In the spirit of reconceptualizing adolescence in the 21st century, in this manuscript we highlight the normative nature of this tension and expand on the idea that young people deserve developmentally appropriate and safe opportunities for enacting maturity (Hoyt et al., 2021). We conclude that whether and how enacting maturity may promote wellbeing and/or confer developmental risk must be examined in light of youth’s structural realities, intersectional identities, as well as their phenomenological processing of their experiences (Velez and Spencer, 2018). Opportunities for enacting maturity should be equitable, scaffolded by adult allies with training, and promoted within responsive organizations. Developmental scientists should focus on understanding and advocating for supportive environments where adolescents from diverse backgrounds can enact maturity in authentic, meaningful, and safe ways.

## Author contributions

PB and LH conceptualized and co-wrote this manuscript. JJ contributed to the conceptualization, literature review, writing, and editing. All authors contributed to the article and approved the submitted version.
